# Screening of small molecules using the inhibition of oligomer formation in α-synuclein aggregation as a selection parameter

**DOI:** 10.1038/s42004-020-00412-y

**Published:** 2020-12-18

**Authors:** Roxine Staats, Thomas C. T. Michaels, Patrick Flagmeier, Sean Chia, Robert I. Horne, Johnny Habchi, Sara Linse, Tuomas P. J. Knowles, Christopher M. Dobson, Michele Vendruscolo

**Affiliations:** 1 Centre for Misfolding Diseases, Department of Chemistry, University of Cambridge, Cambridge, CB2 1EW UK; 2 Paulson School of Engineering and Applied Sciences, Harvard University, Cambridge, MA 02138 USA; 3 Department of Chemistry, Division for Biochemistry and Structural Biology, Lund University, 221 00 Lund, Sweden

**Keywords:** Screening, Biophysical chemistry

## Abstract

The aggregation of α-synuclein is a central event in Parkinsons’s disease and related synucleinopathies. Since pharmacologically targeting this process, however, has not yet resulted in approved disease-modifying treatments, there is an unmet need of developing novel methods of drug discovery. In this context, the use of chemical kinetics has recently enabled accurate quantifications of the microscopic steps leading to the proliferation of protein misfolded oligomers. As these species are highly neurotoxic, effective therapeutic strategies may be aimed at reducing their numbers. Here, we exploit this quantitative approach to develop a screening strategy that uses the reactive flux toward α-synuclein oligomers as a selection parameter. Using this approach, we evaluate the efficacy of a library of flavone derivatives, identifying apigenin as a compound that simultaneously delays and reduces the formation of α-synuclein oligomers. These results demonstrate a compound selection strategy based on the inhibition of the formation of α-synuclein oligomers, which may be key in identifying small molecules in drug discovery pipelines for diseases associated with α-synuclein aggregation.

## Abstract (web summary)

Promising treatments for neurogenerative disorders may involve targeting kinetic intermediates, including α-synuclein oligomers. Here a kinetic method for quantifying oligomer populations is used to screen small molecule inhibitors of oligomerisation and gain mechanistic insight into their modes of action.

## Introduction

α-Synuclein is an intrinsically disordered protein involved in the maintenance of presynaptic vesicle pools, their fusion to plasma membranes and their release from synaptic termini^1–5^. Mutations and duplications of the α-synuclein gene have been implicated in the early onset of Parkinson’s disease^6,7^, a neurodegenerative disorder that affects the motor system^8,9^. This disease is currently incurable, with available drugs capable of improving its symptoms but unable to arrest its onset and progression^10–19^. At the molecular level, this disease, as well as a range of related conditions collectively known as synucleinopathies, is characterised by the presence of deposits predominantly comprised by α-synuclein^20^. Consequently, the inhibition of α-synuclein aggregation has emerged as a strategy for therapeutic intervention for these disorders^12,13,16,21–26^. Despite the focus of research efforts on this target, however, the mechanism of α-synuclein aggregation remains to be fully elucidated. Moreover, there is still a need to develop quantitative experimental methods to enable accurate assessments of the effects of candidate anti-aggregation compounds.

It is also becoming increasingly clear that small, transient oligomeric assemblies, rather than mature, highly ordered fibrils, are highly damaging species associated with α-synuclein aggregation, as they are able to cause, in particular, lipid membrane disruption and cellular toxicity^27–32^. Therefore, it may not be sufficient to inhibit the formation of fibrils, as their measurement does not readily account for the accumulation of damaging oligomeric species^33^. Therefore, compounds that inhibit fibril formation may leave unchanged the prevalence of oligomers or even favour their formation. For this reason, interventions aimed at reducing the reactive flux towards oligomers must take into account the complex interplay between the different microscopic steps of aggregation, as it has been recently shown in the case of the amyloid β peptide^34^.

In the case of α-synuclein, developing drug discovery strategies based on our understanding of its aggregation process is particularly important given that the specific microscopic steps in the aggregation of this protein are highly sensitive to the cellular environment and solution conditions. In particular, the rate of fibril-catalysed secondary nucleation, which is responsible for the rapid proliferation of aggregates, and thus thought to be a major source of toxic α-synuclein oligomers, can change by over four orders of magnitude depending on the pH^35^. As the reactive flux towards oligomeric species depends on a non-linear combination of the various microscopic rates of the constituent aggregation processes, it is challenging to identify compounds that reduce this flux across the range of conditions present in biological systems without employing a method for its accurate quantification as a metric to describe drug efficacy.

In this work, we illustrate a screening strategy to achieve this result. Our approach is based on recent advances in the understanding of the conditions that influence α-synuclein aggregation, such as pH and the effects of varying ratios of seed fibrils to free monomers. This increased understanding has given rise to a set of different experimental assays through which α-synuclein aggregation may be measured by thioflavin T (ThT) fluorescence, which individually recapitulate fibril elongation and secondary nucleation, whereby new aggregates nucleate on existing fibril surfaces. Combined together, these assays enable a detailed characterisation of α-synuclein aggregation process in vitro^35,36^. Crucially, this approach involves a chemical kinetics analysis that enables the quantification of the populations of the intermediate species (i.e. the oligomers) from the measurement of the products (i.e. the fibrils)^37,38^. These experiments occur under quiescent conditions and have been shown to be reproducible under consistent solution conditions^35,36^.

The use of this kinetic strategy has previously enabled the detailed study of α-synuclein aggregation in terms of its microscopic processes under quiescent conditions, providing crucial insights of more direct physiological relevance than those resulting from assays based on shaking of α-synuclein solutions^35^. More specifically, in our approach, the initiation of the aggregation process is studied by means of two seed-induced aggregation assays that recapitulate fibril elongation and fibril amplification. Fibril elongation, which involves the addition of protein monomers to fibril ends, is studied in the presence of high concentrations of preformed fibrils at neutral pH^35,39^ and the autocatalytic amplification of fibrils via secondary nucleation is studied in the presence of low concentrations of preformed fibrils under mildly acidic conditions^35,39,40^.

Our kinetics-based screening approach enables the testing of potential inhibitors for their ability to interfere with the microscopic processes of α-synuclein aggregation and thus with the formation of oligomeric species. We show that this approach can be used to establish a structure–kinetic activity effect^34^ in order to quantify the reduction of α-synuclein oligomers induced by systematic changes to candidate small molecule inhibitors.

## Results

### A kinetics-based drug discovery strategy to target oligomers in synucleinopathies

In this work, we set out to develop a strategy for the identification of small molecules able to interfere with the aggregation process of α-synuclein in a specific manner, whereby the flux towards oligomeric species is reduced.

**Fig. 1:**
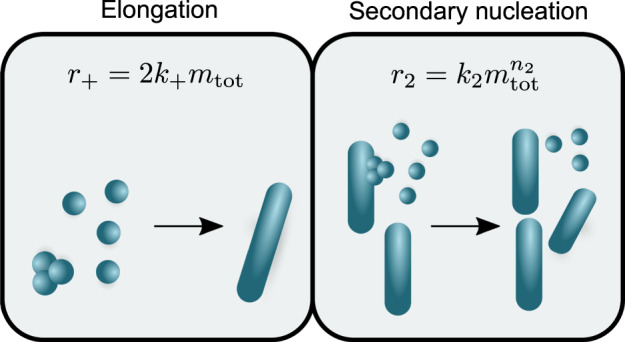
Schematic view of the dominant microscopic processes responsible for the proliferation of α-synuclein oligomers. The inhibition of oligomer proliferation should balance the autocatalytic generation of oligomers by secondary nucleation and the depletion of oligomers by fibril elongation. *r*_+_ is the apparent rate of fibril elongation and *r*_2_ is the apparent rate of secondary nucleation.

Our study consists of a structure–kinetic activity effect analysis of a selected small molecule scaffold, wherein we characterise in detail a series of derivatives against key microscopic steps of α-synuclein aggregation. We thus identify the manner in which different derivatives of this scaffold affect the different microscopic aggregation steps. Specifically, we describe the aggregation process using a master equation approach^41,42^, which quantifies the populations of each aggregating species in terms of the elementary events of aggregation, including fibril elongation (Eq. 1) and secondary nucleation (Eqs. 2–7) (Fig. 1). We concentrate our attention on secondary nucleation in particular, as it has recently been shown that the autocatalytic nature of this process is more effective than primary nucleation in producing oligomeric species^42^. In this master equation framework, we use *k*_+_ and *k*_2_ to denote the rate constants of elongation and secondary nucleation, respectively, while *n*_2_ denotes the reaction order of secondary nucleation.

This master equation approach can be generalised to account for the presence of inhibitors, as it can be shown that the topology of the aggregation network remains unchanged in their presence, i.e. the form of the solution to the aggregation kinetics remains unchanged^33^. This aspect of the approach allows us to interpret inhibited aggregation kinetics in terms of effective rate parameters that are functions of the inhibitor concentration.

**Fig. 2:**
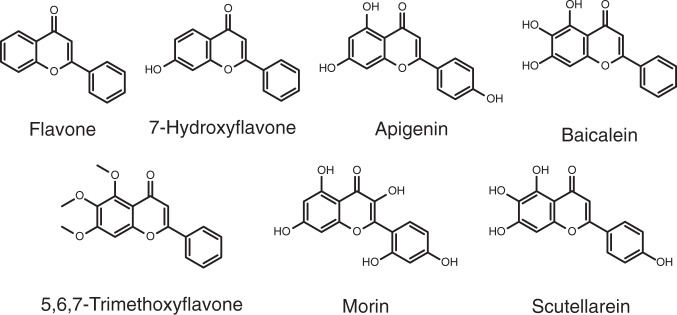
List of the six flavone derivatives tested in this work against the microscopic processes of α-synuclein aggregation. From the flavone scaffold (FLV), we selected 5,6,7-trimethoxyflavone (TMF), 7-hydroxyflavone (7HF), apigenin (API), baicalein (BAI), scutellarein (SCT) and morin (MOR), with varying numbers and positions of hydroxyl moieties.

We initially identified a molecule with the flavone scaffold (FLV; Fig. 2), which affects the aggregation of α-synuclein according to previous reports^43–45^. This molecule is a convenient tool compound for research, and may perhaps be of therapeutic interest, as it has been reported to be neuroprotective in animal models of age-related pathology^46^. We thus employed this molecule to investigate derivatives with varying numbers and positions of hydroxyl moieties. This strategy resulted in the selection of six derivatives for screening (Fig. 2). We note that these compounds may mediate the inhibition of α-synuclein aggregation in a number of ways, both as inhibitors and as promoters, including undergoing an interaction with α-synuclein side chains by forming quinoprotein adducts^47–50^.

### Quantification of reactive flux towards oligomers in α-synuclein aggregation

Oligomeric species produced on the aggregation pathways of disease-associated proteins are often able to induce cytotoxicity to a more severe degree than mature, fibrillar aggregates^27–32^. Indeed, α-synuclein has been shown to form oligomeric aggregates that interconvert between structural subtypes and have varying levels of cytotoxicity mediated by their structures^31,32^. In particular, α-synuclein oligomers possessing a higher degree of cross-β sheet structure, and increased proteinase K resistance, are more harmful in various assays than their less ordered counterparts^31,32^.

Addressing the issue of intermediate species formation from a therapeutic perspective has proven challenging, not least because these species are transient, disordered and difficult to characterise in situ^31,32,37^. However, when abrogating oligomer formation can be considered as a therapeutic target, the need exists to elucidate the rate and quantity of oligomeric species formation as a function of typical fibril-formation kinetic readouts so that, in turn, drug candidates can be screened for their ability to inhibit this process. In particular, the generation of aggregates via secondary nucleation is largely dependent on both the secondary nucleation rate (*r*_2_) as well as the fibril elongation rate (*r*_+_)^34^. These microscopic steps differentially affect the generation of toxic oligomers over time, and thus the prediction of such toxic oligomeric species in the presence of the small molecules requires a simultaneous analysis of both *r*_+_ and *r*_2_ as gathered from the kinetic data^33,34^. While biophysical characterisation of these intermediate species remains challenging, we leverage kinetic parameters in this work to access a predictive model of the flux towards on-pathway oligomeric species.

### Secondary nucleation in α-synuclein aggregation

**Fig. 3:**
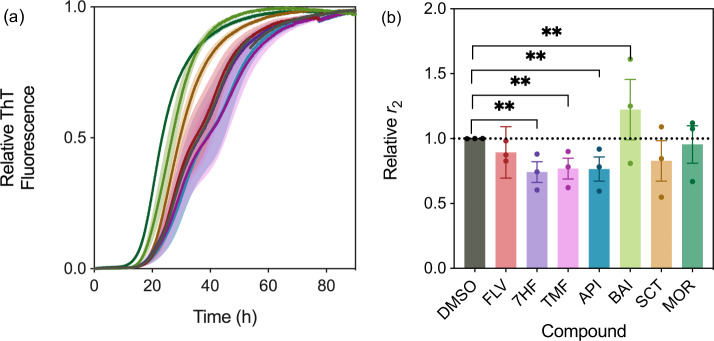
The flavone derivatives affect the α-synuclein secondary nucleation step differentially. **a** Normalised change in ThT fluorescence when 20 µM monomeric α-synuclein was incubated with 50 nM preformed seed fibrils at pH 4.8 and 37 °C in the absence (DMSO control (black)) and presence of 10 µM (0.5 molar equivalents, relative to monomeric protein) of flavone derivatives (flavone (red), 5,6,7-trimethoxyflavone (magenta), 7-hydroxyflavone (purple), apigenin (blue), baicalein (light green, which speeds up the process), scutellarein (brown), morin (dark green)). Yellow overlay indicates corresponding generalised logistic fit (Eq. 2). **b** Rate of α-synuclein fibril amplification, normalised relative to the DMSO control. Error bars represent the standard error of the mean of three experimental replicates each containing three technical replicates. Statistical analyses represent two-way ANOVA results where **P ≤ 0.0021.

We explored first the ability of the derivatives to inhibit secondary nucleation. To this end, we incubated monomeric α-synuclein with 0.0025 molar equivalents of preformed fibrils in the absence and presence of 0.5 molar equivalents of the derivatives, relative to α-synuclein monomers, under mildly acidic conditions^35,36,39^, and analysed the rate of fibril amplification by determining the change of mass fraction of fibrils at the half-time of the aggregation by fitting a generalised logistic function (Eq. 2). Under these conditions, we found that the flavone derivatives exerted differential effects on the aggregation of α-synuclein. In particular, while baicalein and morin were able to accelerate slightly the secondary nucleation process of α-synuclein, other flavone derivatives were shown to inhibit this process instead (Fig. 3). As small molecules may perturb the monitoring of the aggregation process^51^, endpoint fibrils from this reaction were observed by transmission electron microscopy (Fig. S1).

### Elongation of α-synuclein aggregates

**Fig. 4:**
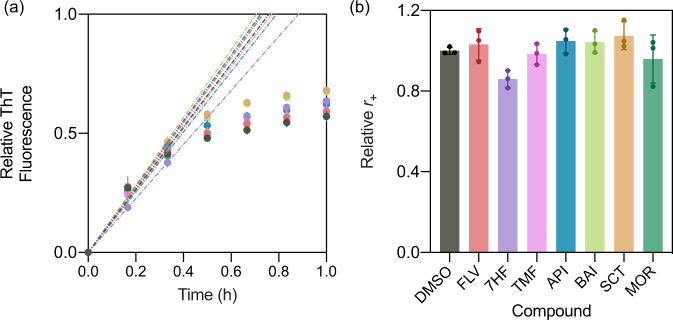
Derivation of the elongation rate constant, *r*_+_, under secondary nucleation conditions. **a** Normalised change in ThT fluorescence when 20 µM monomeric α-synuclein was incubated in the absence (DMSO control (black)) and presence of 0.5 molar equivalents of flavone derivatives (flavone (red), 5,6,7-trimethoxyflavone (magenta), 7-hydroxyflavone (purple), apigenin (blue), baicalein (light green), scutellarein (brown), morin (dark green)) with 30% preformed seed fibrils, relative to monomeric protein, at pH 4.8 and 37 °C under quiescent conditions. Dotted lines indicated the linear fit to the initial time points to obtain *r*_+_. **b** Value of the elongation rate constant, *r*_+_, for each compound, relative to DMSO control. Error bars represent the standard deviation over three technical replicates.

We next investigated the ability of the various flavone derivatives to inhibit α-synuclein fibril elongation. For this purpose, we determine the effective rate of α-synuclein fibril elongation, *r*_+_, under the same conditions used in the secondary nucleation assay (pH 4.8, 37 °C; Fig. 4), in order to garner comparable effective rates for both the elongation and secondary nucleation processes. These conditions were chosen after scanning a range of values to find those suitable for both assays simultaneously. We found that the flavone derivatives only affected mildly the aggregation of α-synuclein (Fig. 4). Thus the inhibitory or accelerating effects as observed in a low-seeded condition is likely to be due to the changes in the secondary nucleation step, rather than the elongation process.

### Using microscopic rates to estimate oligomer formation

**Fig. 5:**
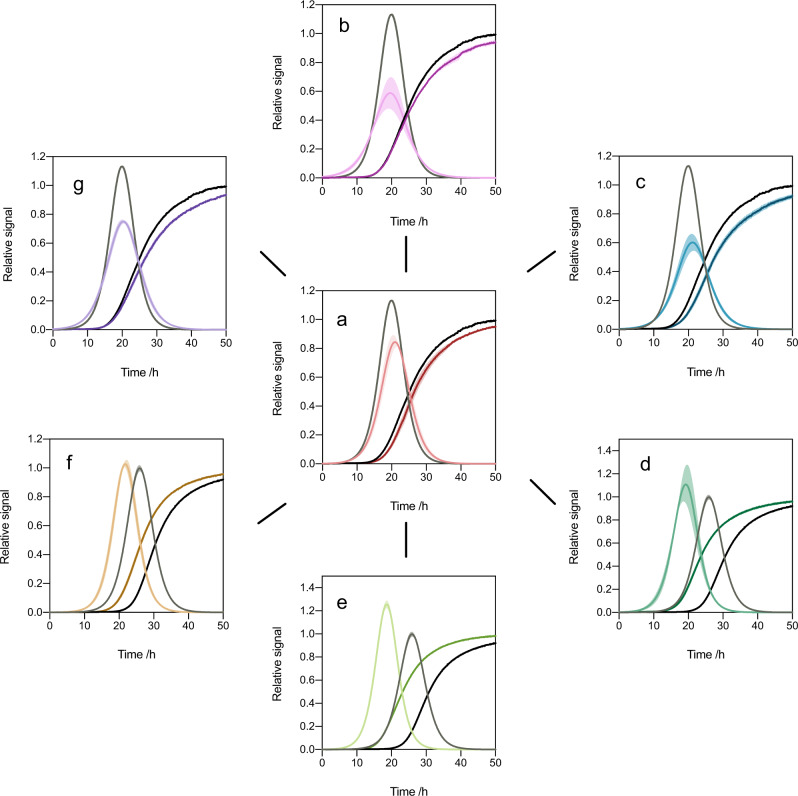
Effects of the flavone derivatives on the reactive flux towards α-synuclein oligomers. **a**–**g** Normalised changes in ThT fluorescence when 20 µM monomeric α-synuclein with 50 nM preformed seed fibrils at pH 4.8 and 37 °C was incubated in the absence (DMSO control (black)) and presence of 10 µM of flavone derivatives, denoted by the dark coloured curve (flavone (**a**), 5,6,7-trimethoxyflavone (**b**), apigenin (**c**), morin (**d**), baicalein (**e**), scutellarein (**f**), 7-hydroxyflavone (**g**)). The normalised reactive flux towards oligomers, *ϕ* (see Eq. 8), which has a characteristic bell shape, is plotted against time and overlaid for each flavone derivative at 0.5 molar equivalents of compound, relative to α-synuclein, denoted by the light grey curve (DMSO control) and the lightly coloured curves.

**Fig. 6:**
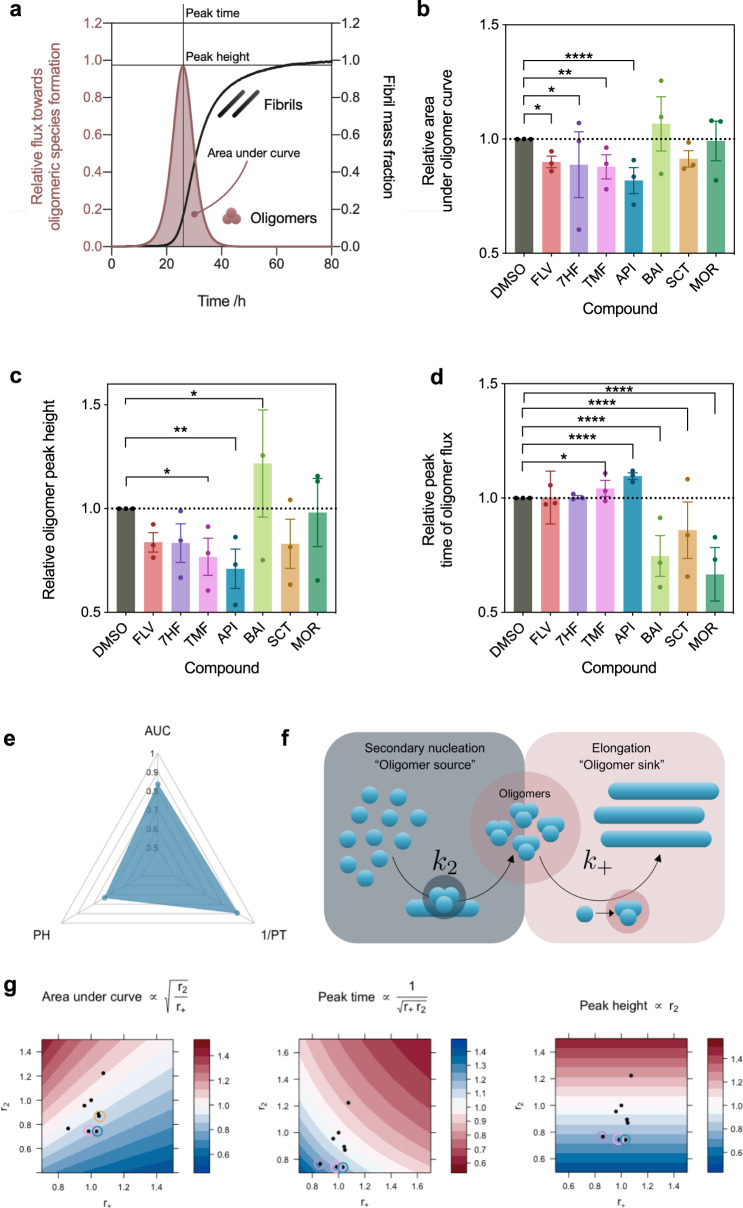
Characterisation of the interplay between the effective rate constants for elongation and secondary nucleation in oligomer formation. **a** General schematic of the time dependence of the oligomer flux in the α-synuclein secondary nucleation assay. **b** Normalised area under the curve of the reactive flux towards α-synuclein oligomer formation relative to the DMSO control. **c** Normalised peak height of the reactive flux towards α-synuclein oligomer formation relative to the DMSO control. **d** Relative peak time of the reactive flux towards α-synuclein oligomer formation relative to the DMSO control. **e** The effect of apigenin on the features of the oligomer flux (*ϕ*), where AUC, PH and PT denote “area under curve”, “peak height” and “peak time”, respectively. **f** Illustration of the manner in which the interplay between secondary nucleation and elongation events determines the oligomer population during aggregation. **g** Visualisation of the interplay of *r*_2_ and *r*_+_ in affecting oligomer flux peak parameters (Eqs. 13–19), with black points indicating the different flavone derivatives. Compounds considered the most effective in each instance are indicated by coloured rings, in particular apigenin (blue), 5,6,7-trimethoxyflavone (pink), 7-hydroxyflafone (purple) and scutellarein (tan). In **b**–**d**, error bars represent the standard error of the mean of three biological replicates each containing three technical replicates. Statistical analyses represent two-way ANOVA results where *P ≤ 0.0332, **P ≤ 0.0021, and ****P ≤ 0.0001.

Using the master equation framework, we derived a mathematical expression for the flux towards oligomers (Eqs. 8–12). Incorporating the effective rates of aggregation into this expression allows us to estimate the effect of different compounds on the flux towards oligomers. Our analysis shows that different compounds have different abilities to affect the flux towards oligomers in the secondary nucleation assay (Figs. 5 and S2). Most notably we observed similar effects on secondary nucleation in the case of apigenin and 7-hydroxyflavone (Fig. 2), but apigenin performed better in decreasing the total area under the oligomer flux curve (that is, the total number of oligomers formed) and decreasing the peak height, as well as in delaying the peak time of the oligomer flux curve (Fig. 6a–e).

Our analysis shows that the flux towards oligomers is not controlled by a single microscopic step of aggregation but rather by a complex combination of different aggregation rates. This conclusion follows if one considers the apparent rate of secondary nucleation (*r*_2_) as a source of new oligomeric intermediates and the apparent rate of elongation of oligomers into fibrillar material (*r*_+_) as a sink that consumes the population of these new aggregates as they grow to mature fibrils (Fig. 6f). Thus the prevalence of oligomeric species in the overall aggregation reaction at any one time is dependent on an interplay between the rates of formation and depletion of oligomers. This interplay dictates a number of characteristic features of the flux towards oligomers over time (area under the curve, peak height and peak time, Fig. 6a–d) and may be visualised, as phase diagrams, in terms of *r*_+_ and *r*_2_ (Eqs. 13–19 and Fig. 6g). The area under the curve, which correlates with the total concentration of oligomers formed, scales as $\sqrt {\frac{{r_2}}{{r_ + }}}$, while the peak time and peak height scale as $1/\sqrt {r_ + r_2}$ and *r*_2_, respectively.

This approach allows us to consider the properties of the resulting oligomer flux curve as parameters through which we can optimise compounds for drug design. Notably, this design cannot be directed towards an optimum for both parameters concurrently. In particular, the optimum design strategies for a compound affecting the peak area or the peak time would involve the optimisation of the effects on both *r*_+_ and *r*_2_, while designing for peak height may involve optimising only inhibition of the secondary nucleation process. These equations may also be generalised to include parameters for fibril fragmentation and dissociation, as well as oligomer conversion^52^. Fibril fragmentation, however, has been observed to be negligible for α-synuclein under quiescent conditions^40^.

## Conclusions

**Fig. 7:**
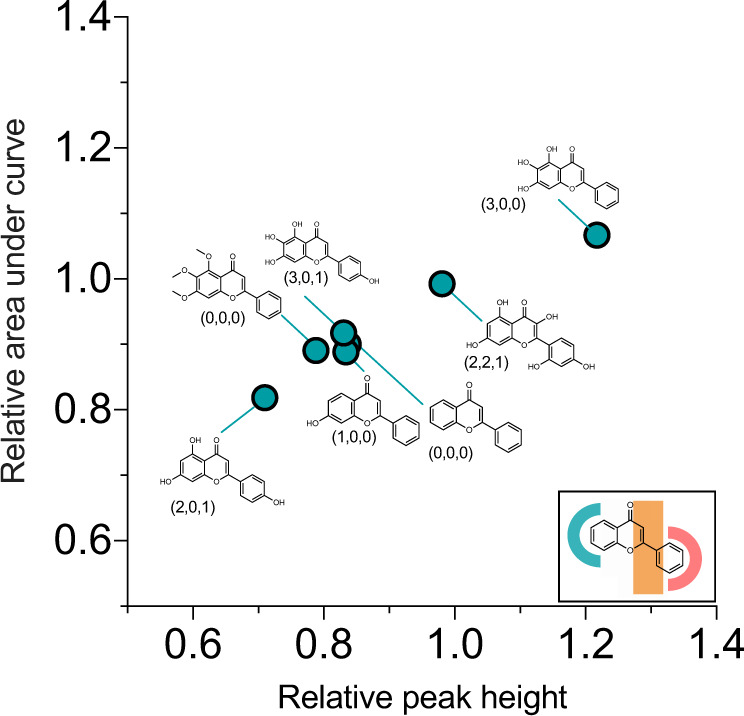
Structure–kinetic activity effect of flavone compounds based on distribution of hydroxyl groups. Compounds are plotted according to their ability to reduce oligomer flux peak height (*x*-axis) and area (*y*-axis). Apigenin (bottom left region of the plot) confers the greatest potency. These findings suggest that the positioning of hydroxyl groups is important for determining the potency of flavone derivatives. More specifically, we define a ‘fingerprint’ for this positioning in terms of the number of hydroxyl groups on the left-hand side, central region and right-hand side (regions denoted in figure inset), respectively, finding that the (2,0,1) fingerprint of apigenin is the most effective one.

We have described an approach that enables the characterisation of the effects of small molecules on the oligomer production via secondary nucleation in α-synuclein aggregation. We have then shown that it is possible to identify a compound (apigenin) that reduces the number of oligomers formed during the aggregation process. The results of a structure–kinetic activity effect study show that the specific positioning of hydroxyl groups on the FLV is important for conferring an enhanced effect (Fig. 7). Our approach is based on a combination of two different assays that makes it possible to study α-synuclein aggregation at low pH values. In future studies, following approaches such as those recently described for Aβ^37^, it will be important to validate the results obtained through this approach with direct measurements of oligomer populations.

These results illustrate how relatively minor chemical modifications can have a significant impact on the microscopic steps in the aggregation process and reveal how the inhibition of oligomer formation should balance oligomer generation by secondary nucleation and oligomer depletion by fibril elongation. Thus the interplay between effects on the respective rates *r*_2_ and *r*_+_ should be carefully considered when a potential lead compound is developed. Overall, our study provides a route for the screening of compounds that reduce the number of α-synuclein oligomers, which can be used as a starting point in drug discovery pipelines.

## Methods

### Reagents

ThT UltraPure Grade (ThT >95%) was purchased from Eurogentec Ltd (Belgium). Sodium phosphate monobasic (NaH_2_PO_4_, BioPerformance Certified >99.0%) and sodium phosphate dibasic (Na_2_HPO_4_, ReagentPlus, >99.0%) were purchased from Merck (formerly Sigma Aldrich), UK.

### Protein preparation

Recombinant α-synuclein was expressed and purified as described previously^35,36,39^. *Escherichia coli* BL21 Gold (DE3) cells were transformed with a human α-synuclein-encoding pT7-7 plasmid and grown in LB (2xγT) media in the presence of ampicillin (100 μg/mL). Cells were induced with 1 mM IPTG, cultured at 37 °C overnight and harvested by centrifugation in a Beckman Avanti J-20 centrifuge with a JA-8.1000 rotor for 20 min at 4000 rpm (Beckman Coulter, Fullerton, CA) and 4 °C. The cell pellet was resuspended in 10 mM Tris–HCl (pH 7.7) and 1 mM EDTA and lysed by sonication. The cell suspension was centrifuged for 20 min at 18,000 rpm at 4 °C, and the supernatant was subsequently boiled by suspension in a water bath at 80–95 °C for 20–25 min. The boiled supernatant was once again centrifuged for 20 min at 18,000 rpm at 4 °C to pellet heat-denatured proteins. In all, 10 mg/mL streptomycin sulfate was added to the supernatant to precipitate DNA and rolled for 15 min at 4 °C on a benchtop rolling system. To pellet precipitated DNA, the mixture was centrifuged at for 20 min at 18,000 rpm at 4 °C and the supernatant was collected. To precipitate α-synuclein, ammonium sulfate was added to the supernatant to yield a final concentration of 361 mg/mL and the mixture was rolled for 30 min at 4 °C on a benchtop rolling system before being centrifuged for 20 min at 18,000 rpm at 4 °C. The α-synuclein-containing pellet was resuspended in 25 mM Tris–HCl (pH 7.7) and dialysed using a 3500 MWCO membrane in 4 L 25 mM Tris–HCl (pH 7.7). α-Synuclein was purified by ion exchange on a Q-Sepharose^TM^ HP HiScale^TM^ 26/20 column (Cytiva, formerly GE Life Healthcare, USA) before size exclusion on a HiLoad^TM^ 16/600 Superdex^TM^ 75 pg column (Cytiva, formerly GE Life Healthcare, USA) into the appropriate experimental buffer. To determine the concentrations in solution, we used the absorbance value of the protein measured at 275 nm and an extinction coefficient of 5600 M^−1^ The protein solutions were divided into aliquots, flash frozen in liquid N_2_ and stored at −80 °C until required for use.

### Seed fibril formation

Seed fibrils were produced as described previously^35,36,39^. Five hundred microlitres of α-synuclein at 100–200 μM concentrations were incubated in 20 mM phosphate buffer (pH 6.5) for 48–72 h at ≈40 °C and stirred at 1,500 rpm with a Teflon bar on an RCT Basic Heat Plate (IKA, Staufen, Germany). Fibrils were divided into aliquots, flash frozen in liquid N_2_ and stored at −80 °C until required for use. For experiments at pH 6.5 (utilising μM fibril concentrations), the fibril stock was sonicated for between 0.5 and 1 min using a probe sonicator (Bandelin, Sonopuls HD 2070, Berlin, Germany), using 10% maximum power and a 50% cycle. For experiments at low pH utilising nM fibril concentrations, the fibril stock was diluted to 10 μM in water and sonicated 3 times for 5 s at 10% maximum power and 50% cycles using the probe sonicator.

### Measurement of aggregation kinetics

Wild-type α-synuclein was incubated at the concentrations indicated and in the presence of 50 μM ThT and preformed α-synuclein fibrils at 37 °C^33,35,39^. The change in the ThT fluorescence signal was monitored using a Fluostar Optima or Polarstar Omega fluorescence plate reader (BMG Labtech, Aylesbury, UK) in bottom reading mode under quiescent conditions. Corning 96-well plates with half-area (3881, polystyrene, black with clear bottom) non-binding surfaces sealed with aluminium sealing tape were used for each experiment.

### Transmission electron microscopy

Wild-type α-synuclein was incubated for 100 h in Corning 96-well plates with half-area (3881, polystyrene, black with clear bottom) non-binding surfaces at the concentrations indicated at 37 °C under quiescent conditions and in the presence of 0.25% preformed fibrils of α-synuclein, as per the secondary nucleation protocol. The endpoint reaction mixture was removed from the plate and adsorbed onto 400 mesh carbon-coated copper grids, stained with 2% aqueous uranyl acetate and visualised with a FEI Tecnai G20 transmission electron microscope, operating at 200 kV, 20 µm objective aperture, and images were recorded with an AMT camera.

### Determination of the fibril elongation rate

In conditions where seed concentrations are high (≃µM), and where primary nucleation can be neglected, the initial rate of aggregate mass build-up can be described as

1 $$\left. {\frac{{{\rm{d}}M(t)}}{{{\rm{d}}t}}} \right|_{t = 0} = 2k_ + P(0)m(0),$$

where *M*(*t*) is the fibril mass concentration at time *t*, *P(*0) is the initial number concentration of fibrils, *m*(0) is the initial monomer concentration and *k*_+_ is the rate of fibril elongation. By fitting a straight line to the early time points of the aggregation reaction *M*(*t*), a value for 2*k*_*+*_*P*(0)*m*(0) can be found^35,36,39^, allowing the initial aggregation rate constants in the presence of the compounds to be calculated and compared.

### Determination of the secondary nucleation rate

For secondary nucleation experiments at low pH with low (≃nM) seed concentrations, the fibril mass fraction *M*(*t*) over time was determined by fitting a generalised logistic function to the normalised aggregation data^53^

2 $$\frac{{M(t)}}{{m_{{\rm{tot}}}}} = 1 - \frac{1}{{\left[ {1 + \frac{a}{c}e^{\kappa t}} \right]^c}},$$

where *m*_tot_ denotes the total concentration of α-synuclein monomers. The terms *a*, *κ* and *c* are fitting parameters, with

3 $$a = \frac{{\lambda ^2}}{{2\kappa ^2}}$$

and

4 $$c = \sqrt {\frac{2}{{n_2\left( {n_2 + 1} \right)}}}.$$

The terms *λ* and *κ* represent combinations of the effective rate constants for primary and secondary nucleation, respectively, and are described as^53^

5 $$\lambda = \sqrt {2k_ + k_nm_{{\mathrm{tot}}}^{n_c}} ,$$

6 $$\kappa = \sqrt {2k_ + k_2m_{{\mathrm{tot}}}^{n_2 + 1}} ,$$

where *k*_*n*_ and *k*_2_ are the rate constants for primary and secondary nucleation, and *n*_*c*_ and *n*_2_ denote the reaction orders of primary and secondary nucleation, respectively. In this case, the parameter *c* was employed as a global fitting parameter for all data sets yielding 0.3, corresponding to a reaction order for secondary nucleation of about *n*_2_ ≃ 4.

The fibril number concentration, *P*(*t*), is subject to amplification and is described by $\frac{{{\rm{d}}P\left( t \right)}}{{{\rm{d}}t}}$, determined in this case at the reaction half-time^39^. Change in α-synuclein fibril concentration under these conditions can be approximated to the change in *P*, described by the change in $\frac{{{\rm{d}}M\left( t \right)}}{{{\rm{d}}t}}$. Thus the model describing the autocatalytic fibril amplification of α-synuclein stems from the linear equation for monomer consumption as described in the case for determining the rate of α-synuclein elongation, *k*_+_, but describes the change in fibril number concentration over time in terms of the total α-synuclein monomer concentration in the reaction, *m*_tot_, and the first derivative of *M*(*t*), described by primary and secondary nucleation processes in Eq. 3, which is then rearranged to give^39^

7 $$\frac{{{\rm{d}}P(t)}}{{{\rm{d}}t}} = \left( {\left. {\frac{1}{{m_{{\rm{tot}}}}} \cdot \frac{{{\rm{d}}m(t)}}{{{\rm{d}}t}}} \right|_{t = t_{1/2}}} \right)^2 \cdot \frac{4}{{k_ + }} ,$$

where and *m*(*t*) is the α-synuclein monomer concentration at the start and at time *t* and $\frac{{{\rm{d}}P(t)}}{{{\rm{d}}t}}$ was determined at the half-time for each reaction and compared, per compound, to the dimethyl sulfoxide control sample to give an effective rate of fibril amplification, *r*_2_^35,36,39^.

### Analysis of on-pathway oligomer formation over time

The theoretical prediction of the time evolution of the reactive flux towards oligomers (Figs. 6a–g and S2), *ϕ*(*t*), was calculated as^39,53^

8 $$\phi (t) = \frac{1}{{r_ + }} \cdot \left[ {\frac{{m(0)}}{{m(t)}} \cdot \frac{{{\rm{d}}^2M}}{{{\rm{d}}t^2}} +\frac{1}{m(0)} \left( {\frac{{m(0)}}{{m(t)}} \cdot \frac{{{\rm{d}}M(t)}}{{{\rm{d}}t}}} \right)^2} \right],$$

where *r*_+_ = 2*k*_+_*m*(0) is the apparent elongation rate constant extracted from the high-seed α-synuclein aggregation experiments for each respective compound (here we use *m(0)* = *m*_*tot*_).

This expression for *ϕ*(*t*) can be derived as follows^39^. The flux towards oligomers is determined by the rate of nucleation, proportional to $\frac{{{\rm{d}}P(t)}}{{{\rm{d}}t}}$. For linearly growing fibrils,

9 $$\frac{{{\rm{d}}M(t)}}{{{\rm{d}}t}} = 2k_ + m(t)P(t) = - \frac{{{\rm{d}}m(t)}}{{{\rm{d}}t}}.$$

Taking the derivative yields

10 $${\frac{{{\rm{d}}^2M(t)}}{{{\rm{d}}t^2}} = 2k_ + \frac{{{\rm{d}}m(t)}}{{{\rm{d}}t}}P(t) + 2k_ + m(t)\frac{{{\rm{d}}P(t)}}{{{\rm{d}}t}}} ={ - \frac{1}{{m(t)}}\left( {\frac{{{\rm{d}}m(t)}}{{{\rm{d}}t}}} \right)^2 + 2k_ + m(t)\frac{{{\rm{d}}P(t)}}{{{\rm{d}}t}},}$$

which can be rearranged to yield

11 $$\frac{{{\rm{d}}P(t)}}{{{\rm{d}}t}} = \frac{1}{{2k_ + m(t)}}\frac{{{\rm{d}}^2M(t)}}{{{\rm{d}}t^2}} + \frac{1}{{2k_ + m(t)^2}}\left( {\frac{{{\rm{d}}m(t)}}{{{\rm{d}}t}}} \right)^2.$$

Substituting Eq. 2 into Eq. 8 yields a description of *ϕ* in terms of the generalised logistic fit parameters as

12 $$\phi (t) = \frac{{{m(0)}a\kappa ^2e^{\kappa t}}}{{2r_ + \left[ {1 + \frac{a}{c}e^{\kappa t}} \right]^2}}$$

from which the area under the curve of *ϕ*, corresponding to the total amount of oligomers formed, may be calculated by the integral

13 $$A = \int _0^\infty {\mathrm{{\phi}}}(t){\rm{d}}t = \frac{{m(0)\kappa c^2}}{{r_ + (a + c)}}.$$

Since

14 $$\kappa \propto \sqrt {r_ + r_2} ,$$

we arrive at an approximation for the area, *A *

15 $$A \propto \frac{{\sqrt {r_ + r_2} }}{{r_ + }} = \sqrt {\frac{{r_2}}{{r_ + }}} .$$

Equation 12 may also be used to calculate the peak height and peak time of *ϕ*, when we consider that the peak time is obtained as a solution to

16 $$\left. {\frac{{{\rm{d}}{\mathrm{{\phi}}}}}{{{\rm{d}}t}}} \right|_{t = t_{{\mathrm{peak}}}} = 0 \Rightarrow t_{{\mathrm{peak}}} = \frac{1}{\kappa }{\mathrm{ln}}\left( {\frac{c}{a}} \right),$$

which yields a description for the peak height of *ϕ* as

17 $${\mathrm{{\phi}}}_{{\mathrm{peak}}} = {\mathrm{{\phi}}}\left( {t_{{\mathrm{peak}}}} \right) = \frac{{m(0)\kappa ^2c}}{{4r_ + }}.$$

Using Eqs. 16 and 17, in combination with Eq. 14, we arrive at

18 $$t_{{\mathrm{peak}}} \propto \frac{1}{{\sqrt {r_ + r_2} }}$$

and

19 $${\mathrm{{\phi}}}_{{\mathrm{peak}}} \propto \frac{{r_ + r_2}}{{r_ + }} = r_2$$

as scaling relationships for peak time and peak height, respectively. Thus the parameters of area under the curve, peak height and peak time may all be approximated in terms of the fitting parameter *κ*, which, in turn, represents a combination of the effective elongation rate, *r*_+_, and the effective secondary nucleation rate, *r*_2_.

## Supplementary information

Supplementary Information

## Data Availability

The data that support this study are available from the corresponding author upon reasonable request.
