# Supplementary material for: Screening of small molecules using the inhibition of oligomer formation in α-synuclein aggregation as a selection parameter
Source: Commun Chem. 2020 Dec 18;3:191. doi: 10.1038/s42004-020-00412-y (PMC9814678; doi:10.1038/s42004-020-00412-y)
Supplement: Supplementary file 1 — Supplementary Information [file 42004_2020_412_MOESM1_ESM.pdf]

Supplementary Information

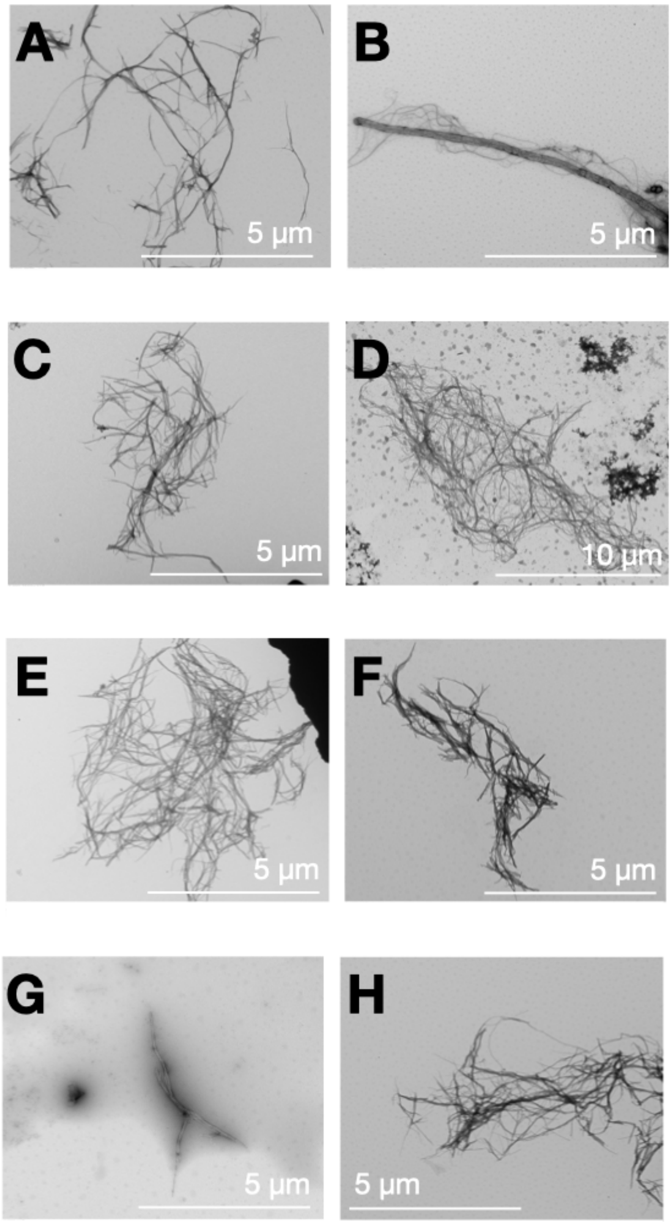

Figure S1. Transmission electron microscopy (TEM) of α-synuclein secondary nucleation reaction endpoints. TEM images of the endpoint fibrils formed during α-synuclein secondary nucleation when 20 µM monomeric α-synuclein was incubated in the absence (DMSO control (A)) and presence of 0.5 molar equivalents, relative to monomeric protein, of flavone derivatives (flavone (B), 7-hydroxyavone (C), 5,6,7-trimethoxy (D), apigenin (E), baicalein (F), scutellarein (G), morin (H)) with 50 nM preformed seed fibrils at pH 4.8 and 37 °C.

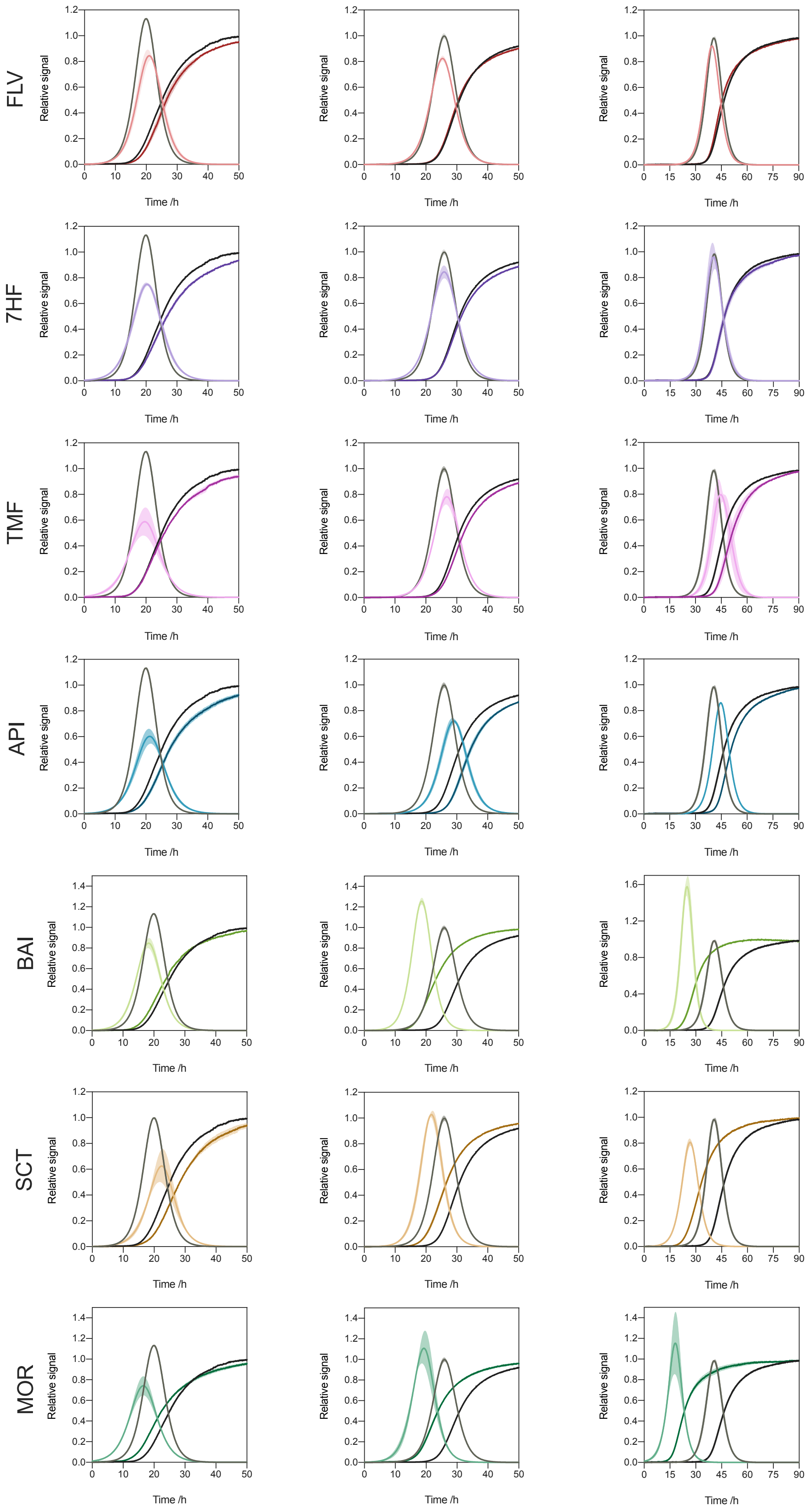
FLV
7HF
TMF
API
BAI
SCT
MOR
Relative signal
Time /h

Figure S2. Effects of flavone derivatives on the reactive flux towards α-synuclein oligomers in the secondary nucleation assay. Normalised changes in ThT fluorescence (sigmoidal curves) in α-synuclein secondary nucleation in vitro assays with 50 nM preformed seed fibrils at pH 4.8 and 37 °C when 20 μM monomeric α-synuclein was incubated in the absence (DMSO control, black) and presence of 0.5 molar equivalents relative to the total protein, of flavone derivatives: flavone (red), 7-hydroxyflavone (purple), 5,6,7-trimethoxyflavone (magenta), apigenin (blue), baicalein (light green), scutellarein (tan), morin (dark green). The corresponding normalised reactive fluxes towards oligomers, ϕ (peaked curves, see **Eq. 8**), are plotted against time and overlaid for each flavone derivative. Each plot represents three experimental replicates, while the three different plots per molecule represent biological replicates.
